# Editorial: Integrating Neurophysiological and Behavioral Changes From Midlife to Old Age

**DOI:** 10.3389/fnagi.2022.964473

**Published:** 2022-06-28

**Authors:** Maayan Agmon, Shawn D. Youngstedt, Tamar Shochat

**Affiliations:** ^1^The Cheryl Spencer Department of Nursing, Faculty of Social Welfare and Health Sciences, University of Haifa, Haifa, Israel; ^2^The Cheryl Spencer Institute of Nursing Research, Faculty of Social Welfare and Health Sciences, University of Haifa, Haifa, Israel; ^3^Edson College of Nursing and Health Innovation, Arizona State University, Phoenix, AZ, United States

**Keywords:** aging, multifactorial etiology, midlife, early biomarkers, interdisciplinary approach, prevention

The global rise of the older population has led to widespread scientific interest in the aging process. This interest consists of two complementary goals: (1) to gain a better understanding of human development; (2) to promote healthy aging while reducing the burden often involved in growing older. The study of aging encompasses a wide diversity of fields, including biology, physiology, psychology, and sociology. However, a common limitation is that research generally originates from an individual discipline rather than adopting an interdisciplinary approach. Consequently, our understanding of the various mechanisms underlying the aging processes is restricted. Furthermore, there has been an inadequate focus on the early manifestations of the aging process which predate older age. In the present issue of *Frontiers in Aging Neuroscience*, we searched for articles that bridged these gaps. The academic papers in this Research Topic, *Integrating Neurophysiological and Behavioral Changes From Midlife to Old Age*, shed light on the multifactorial nature of the aging process and cover a broad spectrum of topics including behavioral, brain structural and physiological levels, ranging from midlife to old age.

A major priority in geroscience is identifying early markers of normal or accelerated aging processes. As a result, these papers have identified markers of aging, such as reticulospinal tract connectivity, low-grade inflammation, cardiovascular fitness level, and caregiver strain (or burden), which predict early aging manifestations, including muscle weakness as a proxy marker for sarcopenia, gait performance, brain white matter integrity, and sleep disturbances. These particular factors have previously been linked to accelerated aging, neurodegenerative diseases (e.g., Alzheimer's dementia and Parkinson's disease), as well as increased cardiometabolic risk.

Study designs in this issue include one longitudinal study spanning over 20 years (Heumann et al.), one cross-sectional secondary analysis of the Dunedin Longitudinal Study (d'Arbeloff et al.), and two cross-sectional designs (Osakwe et al.; Maitland and Baker). Although some of the instruments are widely used and well validated in studies of older adults (e.g., grip strength and gait performance under dual-task conditions), evidence for their relevance and predictive validity among middle age adults is scant. For example, although gait under single- and dual-task conditions is a well-established marker for neurodegenerative diseases and accelerated aging in older age, evidence for its predictive value in midlife is currently weak.

The above four studies have the potential to contribute both in theory and practice. From a theoretical point of view, these studies indicate the importance of stronger longitudinal designs, the incorporation of a multidisciplinary approach, and the importance of establishing the predictive value of the collection of early markers during midlife.

These research papers carry important implications for health practices and provide some intriguing directions for future aging research. For example, Maitland and Baker argue that interventions which reinforce the reticulospinal tract, such as a startle reflex to acoustic stimuli, could provide a treatment or prevention of age-related sarcopenia. Heumann et al.'s novel findings suggest that targeting inflammation at an early age may alleviate gait decline and related sequelae in the course of aging. The d'Arbeloff et al. paper adds to an extensive and growing quantity of literature suggesting the importance of aerobic fitness and physical activity in preventing cognitive impairment and reducing the risk of Alzheimer's disease and related dementias. Finally, Osakwe et al. remind us that sleep-improvement interventions can be helpful in reducing cardiovascular risk in the growing numbers of *caretakers* of individuals with dementia. To this end, collaborative efforts should focus on the implementation of interdisciplinary research in practice in order to promote an active and meaningful aging process for the broader population.

## Author Contributions

MA, SDY, and TS contributed to the conceptualization, writing, and editing of the editorial. All authors contributed to the article and approved the submitted version.

## Conflict of Interest

The authors declare that the research was conducted in the absence of any commercial or financial relationships that could be construed as a potential conflict of interest.

## Publisher's Note

All claims expressed in this article are solely those of the authors and do not necessarily represent those of their affiliated organizations, or those of the publisher, the editors and the reviewers. Any product that may be evaluated in this article, or claim that may be made by its manufacturer, is not guaranteed or endorsed by the publisher.

